# *Leishmania donovani*-induced expression of signal regulatory protein α on Kupffer cells enhances hepatic invariant NKT-cell activation

**DOI:** 10.1002/eji.200939863

**Published:** 2010-01

**Authors:** Lynette Beattie, Mattias Svensson, Alison Bune, Najmeeyah Brown, Asher Maroof, Soombul Zubairi, Katharine R Smith, Paul M Kaye

**Affiliations:** Centre for Immunology and Infection, Hull York Medical School and Department of Biology, University of YorkYork, UK

**Keywords:** CD47, Invariant NKT cells, Kupffer cells, Signal regulatory protein α

## Abstract

Signal regulatory protein α (SIRPα) and its cognate ligand CD47 have been documented to have a broad range of cellular functions in development and immunity. Here, we investigated the role of SIRPα–CD47 signalling in invariant NKT (iNKT) cell responses. We found that CD47 was required for the optimal production of IFN-γ from splenic iNKT cells following exposure to the αGalCer analogue PBS-57 and *in vivo* infection of mice with *Leishmania donovani*. Surprisingly, although SIRPα was undetectable in the liver of uninfected mice, the hepatic iNKT-cell response to infection was also impaired in CD47^−/−^ mice. However, we found that SIRPα was rapidly induced on Kupffer cells following *L. donovani* infection, *via* a mechanism involving G-protein-coupled receptors. Thus, we describe a novel amplification pathway affecting cytokine production by hepatic iNKT cells, which may facilitate the breakdown of hepatic tolerance after infection.

## Introduction

Signal regulatory protein α (SIRPα, CD172a), also known as Src homology 2 domain-containing phosphatase substrate 1, p84 protein, brain Ig-like molecule with tyrosine based activation motifs, macrophage fusion receptor and Myd-1 1, has multiple functions in immunity and development, linked to its restricted cellular distribution 2,3. The most comprehensive description of the distribution of SIRPα is in the rat, where expression was shown on neurons, monocytes, granulocytes, tissue macrophages and DC 2. In immunity, SIRPα impacts on allogeneic MLR 4, DC maturation and cytokine production 5,6, the activation of memory T cells 7, macrophage cytokine production 8,9 and macrophage fusion 10.

CD47 (also known as integrin-associated protein (IAP)) is the only identified cellular receptor for SIRPα 11. In contrast to the monogamous binding of SIRPα to CD47, CD47 also binds thrombospondin 12,13. *In vitro* CD47 can co-stimulate T cells 14,15, and the effects of CD47 deficiency on human neutrophil transmigration are readily apparent 16 and are similarly observed in rodent models of peritonitis 17, *Staphylococcus aureus* induced arthritis 18 and *Escherichia coli* pneumonia 19. CD47 was recently shown to be a key signal in the development of Th17-mediated experimental colitis *via* interactions with SIRPα on CD103^−^ DC 20, and has shown experimental potential as an immunotherapeutic target for adult leukaemia 21,22.

CD1d-restricted invariant NKT cells (iNKT) play important roles in cancer and infectious disease (for review see 23). In the spleen, DC are crucial for presenting CD1d-restricted ligands to iNKT cells, whereas in the liver, Kupffer cells (KC) 24 and Ito cells 25 perform this function. Although studies on the long-term outcome of *Leishmania donovani* infection suggest that NKT cells may ultimately be redundant in terms of regulating disease progression 26, we have nevertheless previously shown that hepatic iNKT-cell-derived IFN-γ is essential for sustained CXCL10 responses following *L. donovani* infection 27. As Src homology 2 domain-containing phosphatase substrate 1-mutant mice have an impaired ability to clear transferred tumour cells, and lower levels of iNKT-cell cytokine production 28, and as a role for SIRPα–CD47 signalling in iNKT-cell responses to infection has yet to be established, we sought to determine whether this pathway might be involved in regulating early iNKT-cell responses to *L. donovani*. Here, we show regulated expression of SIRPα on KC following *L. donovani* infection and propose that SIRPα–CD47 interactions regulate the activation threshold for iNKT cytokine production.

## Results and discussion

### CD47 regulates iNKT-cell activation

**Figure 1:**
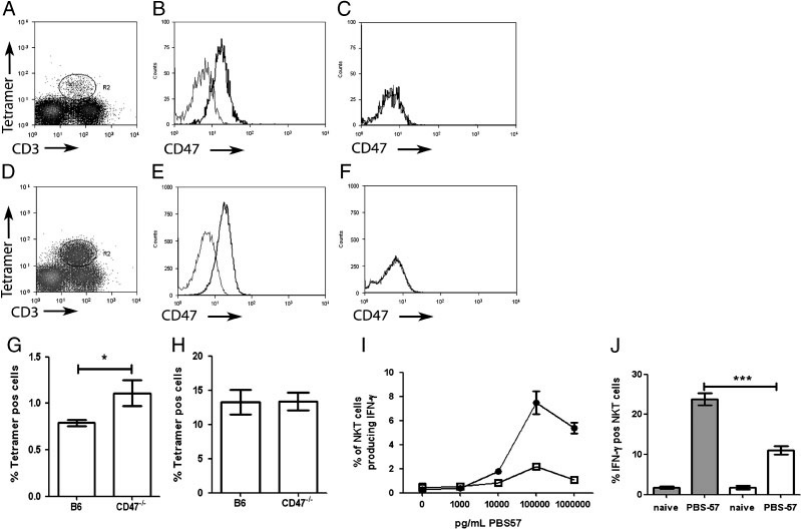
Phenotype, frequency and responsiveness of iNKT cells from CD47^−/−^ mice. (A–F) Expression of CD47 on iNKT cells on spleen (A–C) and liver (D–F) iNKT cells. Similar profile of CD3 and tetramer staining were obtained for CD47^−/−^ mice. Spleen (A) and liver (D) iNKT cells were identified by excluding autofluorescent cells and then gating on PBS-57-loaded CD1d tetramer^+^ CD3^+^cells. Splenic (B) and hepatic (E) iNKT cells in B6 mice express CD47, whereas splenic (C) and hepatic (F) iNKT cells in CD47^−/−^ do not. Dotted lines represent isotype controls. (G–H) The percentage±SEM of tetramer^+^ cells in the spleen (G) and liver (H) of naïve B6 and CD47^−/−^ mice. (*n*=20 individual mice from three independent experiments.) (I) IFN-γ production by splenic tetramer^+^ TCR-β^+^ cells after 16 h *in vitro* stimulation with PBS-57; C57BL/6 (closed circles) and CD47^−/−^ mice (open squares). Data represent mean±SEM of triplicate samples pooled from three to five mice and are representative of three independent experiments. (J) IFN-γ production by splenic tetramer^+^ TCR-β^+^ iNKT cells 16 h after i.v. injection of 10 ng PBS-57. Data represent mean±SEM (*n*=8 mice from two independent experiments). ^*^p<0.01, ^***^p<0.0001, Mann–Whitney *U* test.

As anticipated 28, PBS-57-loaded CD1d tetramer^+^CD3^+^ cells in the spleen and liver of C57BL/6 mice expressed CD47, whereas no detectable staining was observed in CD47^−/−^ mice (Fig. 1A–F). iNKT cells were present at a higher frequency in the spleen, but not the liver of CD47^−/−^ mice when compared with WT controls (Fig. 1G and H). *In vitro* stimulation of splenocytes from CD47^−/−^ and C57BL/6 mice with PBS-57, an analogue of αGal-Cer 29, demonstrated that CD47 was required for optimal production of IFN-γ by iNKT cells, measured as percentage of responding cells or as integrated MFI (iMFI) 30( (Fig. 1I and data not shown). Similarly, injection of PBS-57 stimulated a greater IFN-γ response in WT mice compared with CD47^−/−^ mice (Fig. 1J). Therefore, CD47 is required for optimal production of IFN-γ by iNKT cells in the spleen, where myeloid cells express the CD47 ligand SIRPα constitutively at high levels 2.

### CD47 co-stimulates IFN-γ production by iNKT cells after *L. donovani* infection

**Figure 2:**
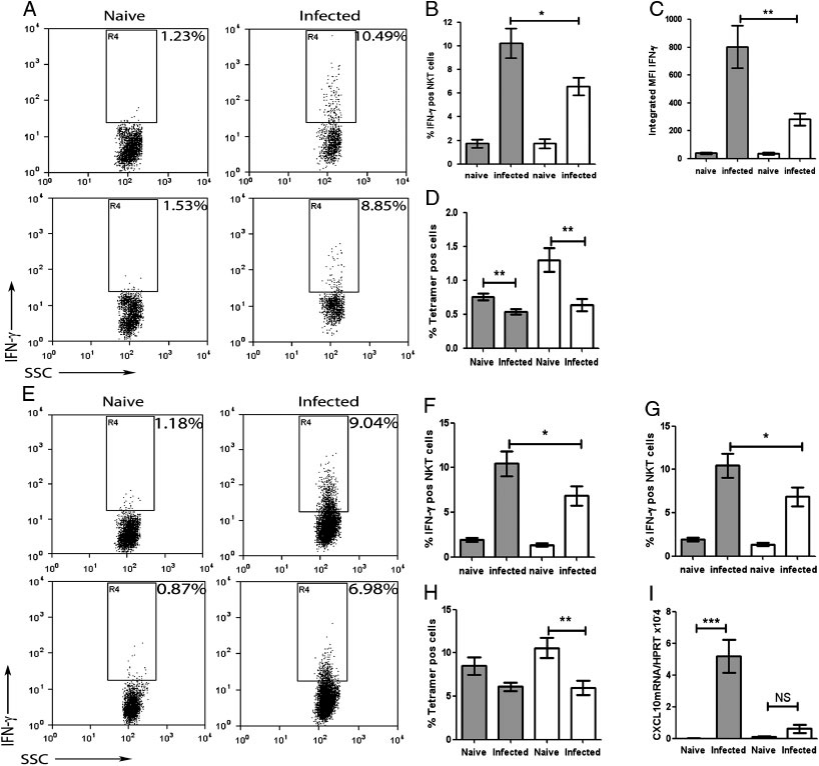
IFN-γ production by iNKT cells is impaired in CD47^−/−^ mice. B6 and CD47^−/−^ mice were infected with *L. donovani* i.v. 16 h previously. Representative dot plots showing IFN-γ production by (A) spleen and (E) liver iNKT cells (based on tetramer/TCR-β and exclusion of autofluorescence) are shown. IFN-γ responses of splenic (B and C) and hepatic (F and G) iNKT cells from naïve and infected B6 (grey bars) and CD47^−/−^ (open bars) mice (*n*=12 from two independent experiments) are shown as percentage of IFN-γ^+^cells (corrected for isotype staining; B and F) and as iMFI (C and G). The frequency of iNKT cells in spleen (D) and liver (H) of naïve and infected B6 (grey bars) and CD47^−/−^ (open bars) mice. (I) CXCL10 mRNA accumulation at 5 h p.i. in B6 (grey bars) and CD47^−/−^ (open bars) mice (*n*=5 mice). ^*^p<0.05, ^**^p<0.01, ^***^p<0.0001, Mann–Whitney *U* test.

*L. donovani* infection results in iNKT-cell activation and IFN-γ production 26,31. To determine whether CD47 also co-stimulated this response, we examined infected WT and CD47^−/−^ mice (Fig. 2). The percentage of splenic tetramer^+^ TCR-β^+^ cells that produced IFN-γ (Fig. 2A and B) and the total functional IFN-γ response (Fig. 2A and C) was significantly reduced in infected CD47^−/−^ mice compared with the WT mice. Reduced detection of IFN-γ-producing cells did not reflect enhanced internalisation of TCR in CD47^−/−^ mice, as similar results were obtained on staining for surface or surface and intracellular TCR (Supporting Information Fig. 1). There was also a reduction in the frequency of splenic iNKT cells after infection (Fig. 2D). CD69 expression in both WT and CD47^−/−^ mice was, however, increased to a similar extent (from an MFI of 44.5±5.6 to 68.4±22, and 42.5±5.4 to 70.6±18.6 in WT and CD47^−/−^ mice, respectively), suggesting similar levels of activation as assessed by this parameter. The expression of CD47 on iNKT cells was unaltered at 16 h post-infection (p.i.) compared with levels seen in uninfected mice (data not shown and Fig. 1).

If, as suggested by the above data, SIRPα–CD47 interactions play a role in iNKT-cell activation, tissue-specific expression of SIRPα might dictate the extent to which this co-stimulatory pathway operates. We therefore examined responses in the liver, where SIRPα expression is reported as low or absent 2. Surprisingly, IFN-γ production by hepatic iNKT cells was also significantly impaired in CD47^−/−^ mice compared with WT mice (Fig. 2E–G). As in the spleen, the frequency of hepatic iNKT cells was reduced in infected CD47^−/−^ mice (Fig. 2H). In contrast to the spleen, however, increased expression of CD69 was limited to iNKT cells in WT mice (from an MFI of 48.1±7.08 to 65.6±12.95) and was not observed on hepatic iNKT cells in CD47^−/−^ mice (MFI of 51.68±5.52 to 58.67±−7.15). These data suggest that CD47 signalling variably affects different parameters of iNKT-cell activation in a tissue-specific manner, with a greater overall dependency on SIRPα–CD47 for hepatic responses.

To determine whether the reduced IFN-γ response of CD47^−/−^ mice was functionally relevant, we measured the accumulation of CXCL10 mRNA, previously shown to be dependent upon iNKT-cell-derived IFN-γ 27. *L. donovani* infection resulted in a rapid accumulation of CXCL10 mRNA in WT but not in CD47^−/−^ mice (Fig. 2I). The reduction in IFN-γ arising from CD47-deficiency, therefore, has measurable down-stream effects on the host response to infection.

### *L. donovani* induces SIRPα expression on KC

**Figure 3:**
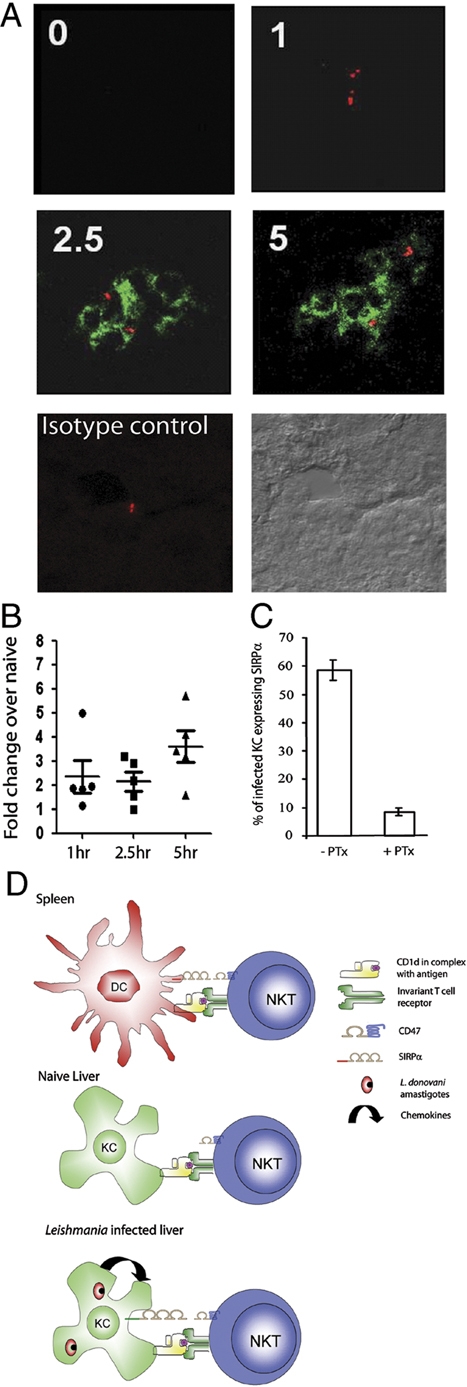
*L. donovani* induces expression of SIRPα on KC. (A) Livers from naive B6 mice or mice infected with *L. donovani* 1, 2.5 or 5 h previously were stained for SIRPα (green) and *L. donovani* AM (red). Images are representative of four independent experiments. (B) Hepatic SIRPα mRNA accumulation, shown as fold increase relative to naïve mice at timepoints indicated. (C) SIRPα expression on KC in control and PTx-treated mice infected with *L. donovani*. Data in (B) and (C) are representative of two independent experiments. (D) Proposed model for regulation of APC–iNKT-cell interactions through CD47-SIRPα signalling. On splenic APC, constitutively expressed SIRPα engages CD47 and enhances the TCR-dependent IFN-γ response of iNKT cells. In the liver, SIRPα is not constitutively expressed on KC, but expression can be induced in response to chemokines produced as a result of *L. donovani* infection.

As SIRPα is the only identified cellular receptor for CD47, yet we detected an impaired hepatic response in CD47^−/−^ mice, we examined expression of SIRPα before and after *L. donovani* infection. Similar to that reported in the rat 2, SIRPα was undetectable on KC in naive mice, but was rapidly induced following infection (Fig. 3A). SIRPα mRNA accumulation also increased following infection, significantly so by 5 h p.i. (*p*<0.05 comparing ΔCT (CT, cycle threshold) values to naïve mice; Fig. 3B). Of all F4/80^+^ KC, 80±1% with clearly visible intracellular amastigotes (AM) were SIRPα^+^, whereas 60±3% of SIRPα^+^ cells had identifiable AM, suggesting that SIRPα was also induced in trans on uninfected KC. Infected SIRPα^+^ cells were not labelled with CD11b, a marker of inflammatory monocytes and neutrophils (data not shown).

We next sought to determine the mechanism(s) responsible. SIRPα expression was similarly induced on KC in infected BALB.SCID and B6.RAG1^−/−^ mice and in B6.IFN-γ^−/−^ and B6.IL-12p40^−/−^ mice (data not shown), suggesting that neither T cells, B cells nor iNKT cells, nor these key pro-inflammatory cytokines were required for SIRPα induction. Parasite viability was not an important factor, as injection of heat killed *L. donovani* also induced SIRPα (data not shown). To address whether other signals could induce SIRPα, we injected mice with (i) latex beads, to reflect the consequences of phagocytosis *per se*; (ii) zymosan, to reflect phagocytosis coupled with stimulation through TLR2 and TLR6 32 and (iii) Poly I:C, as a soluble TLR3 agonist that induces Type I IFN responses 33). None of these stimuli induced SIRPα expression (data not shown). As *L. donovani* infection stimulates a rapid T-cell-independent expression of CCL2, CCL3 and CXCL10 34, and our data suggested that regulation of SIRPα could occur in trans, we used pertussis toxin (PTx) to block G-protein-coupled signalling. Administration of PTx inhibited SIRPα induction by approximately 85% (Fig. 3C), suggesting that G-protein signalling was indeed an essential pre-requisite for the induction of SIRPα following *L. donovani* infection.

## Concluding remarks

Collectively, these data provide the first demonstration of pathogen-associated induction of SIRPα on KC *in vivo.* Our data suggest a model whereby SIRPα on KC is regulated by autocrine or paracrine responses to chemokines released upon infection. In turn, we propose that induction of SIRPα regulates optimal activation of iNKT cells by engagement of CD47 (Fig. 3D) and thus indirectly affects the down-stream progression of the inflammatory response. IL-12 has also been shown to facilitate activation of iNKT cells in conjunction with TLR9 signalling 35 and TLR9-dependent IL-12 production by DC has also been noted following infection with *L. infantum* 36. However, as SIRPα–CD47 signalling inhibits DC maturation and IL-12 production 6, regulation of DC IL-12 *per se* is unlikely to account for the defective iNKT-cell activation we have observed in CD47^−/−^ mice. Further studies will be required to ascertain the long-term impact of disrupting SIRPα–CD47 interactions for the progression of experimental visceral leishmaniasis, and to determine the breadth of infections in which regulated expression of SIRPα may similarly provide a mechanism for breaking hepatic tolerance.

## Materials and methods

### Mice and parasites

BALB/c mice were obtained from Charles River (Margate, UK). C57BL/6 (B6), BALB.SCID, B6.RAG1^−/−^, B6.IFN-γ^−/−^, B6.IL-12p40^−/−^ (originally obtained from the Jackson Laboratories, Bar Horbor, USA) and B6.CD47^−/−^ (originating from breeding pairs supplied by Dr. E. Brown, University of California San Francisco 17) were bred under barrier conditions at LSHTM and the University of York Biological Services Facility. All animal procedures were approved by institutional Animal Procedures Ethics Committees and performed under UK Home Office licence.

*L. donovani* (strain LV9) were isolated from infected hamsters or B6.RAG1^−/−^ mice as previously described 37. Mice were infected with 2–3×10^7^ AM i.v. by the lateral tail vein. In some experiments, an equivalent number of heat-killed AM (56°C for 30 min), zymosan (Sigma-Aldrich, Poole, UK) or latex beads (3 μm; Sigma-Aldrich) were injected. PTx treatment was performed as previously described 38. 10 ng/mouse of PBS-57 (supplied by Paul Savage, Brigham Young University, Provo, UT, USA) was injected i.v. as previously described 29.

### Flow cytometry and intracellular cytokine staining

Splenic and hepatic mononuclear cells were isolated as previously described 26,37. Isolated cells were incubated directly and without further stimulation in brefeldin A (10 μg/mL) for 4 h. Cells were labelled with CD16/32, NK1.1-PE, TCR-β-APC or FITC (eBioscience, UK), CD3-PeCy7 (Biolegend, San Diego, USA) and Alexa-488 or APC conjugated-PBS-57 loaded CD1d tetramers (National Institutes of Health, National Institute of Allergy and Infectious Diseases MHC Tetramer Core Facility). Labelled cells were fixed, permeabilised and labelled with Pacific blue-conjugated IFN-γ or isotype control (eBioscience). Flow cytometric analysis was performed on a CyAn flow cytometer with Summit software (Beckman Coulter, Fullerton, USA). Autofluorescent events were excluded from analysis by gating on unused fluorescent channels. iMFI were calculated by multiplying the frequency of IFN-γ-producing cells by the MFI of the positive population to determine the total functional IFN-γ response 30.

### Histological analysis of SIRPα expression

Livers from infected mice were snap frozen in isopentane, embedded in OCT and stored at −70°C until use. 6 μm cryosections were fixed in acetone and labelled with rat anti-murine p84 biotin antibody (a gift from Carl Lagenaur, University of Pittsburgh), CD11b and F4/80 (eBioscience). AM of *L. donovani* were identified using serum from *L. donovani*-infected hamsters. Images were captured as 0.8–1 μm optical slices using a LSM510 confocal microscope and processed using LSM Image Browser (Zeiss,Jera, Germany).

### Real-time RT-PCR

Real-time RT-PCR was performed as previously described 37. Oligonucleotides used for the specific amplification of SIRPα were CCTCACAGCAACGAAGAACA (forward) and TGGACTCATTCATGGTGCAG (reverse), and for amplification of CXCL10 and hypoxanthine phosphoribosyltransferase (HPRT) were as described previously 37. The number of SIRPα and HPRT cDNA molecules in each sample was calculated using QuantiTect SYBR green master mix (QIAGEN) and an ABI Prism 7000 sequence detection system (Applied Biosystems). Accumulation of *SIRP*α and *Cxcl10* was normalised to HPRT and expressed as either absolute copy number (target molecules/1000 *Hprt* molecules) or relative expression *via* the change in cycle threshold (ΔΔCT) analysis method (relative expression in infected *versus* naïve).

### Statistical analysis

Statistical analysis was performed using two-tailed Mann–Whitney *U* tests with 95% confidence intervals.

## Abbreviations

AM: amastigotes
HPRT: hypoxanthine phosphoribosyltransferase
iMFI: integrated MFI
iNKT: invariant NKT
KC: Kupffer cells
p.i.: post-infection
PTx: pertussis toxin
SIRPa: signal regulatory protein a
