# Supplementary material for: Leishmania donovani-induced expression of signal regulatory protein α on Kupffer cells enhances hepatic invariant NKT-cell activation
Source: Eur J Immunol. 2010 Jan;40(1):117–23. doi: 10.1002/eji.200939863 (PMC2909397; doi:10.1002/eji.200939863)
Supplement: Supplementary file 1 [file eji0040-0117-SD1.pdf]

European Journal of Immunology

Supporting Information

for

DOI 10.1002/eji.200939863

***Leishmania donovani*-induced expression of signal regulatory protein α on Kupffer cells enhances hepatic invariant NKT-cell activation**

Lynette Beattie, Mattias Svensson, Alison Bune, Najmeeyah Brown, Asher Maroof, Soombul Zubairi, Katharine R. Smith and Paul M. Kaye

© 2009 WILEY-VCH Verlag GmbH & Co. KGaA, Weinheim

Beattie et. al. Supporting Information

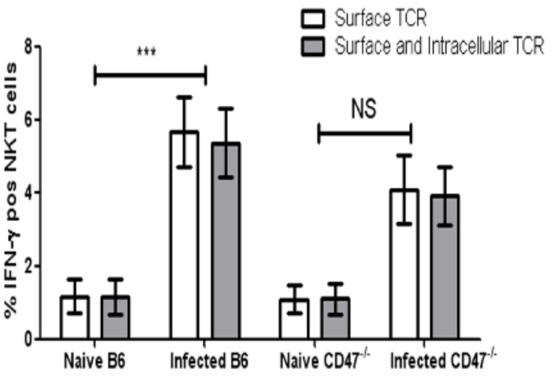

Figure S 1. Detection of iNKT cells in CD47$^{-/-}$ mice is not impaired due to enhanced internalisation of TCR.

B6 and CD47$^{-/-}$ mice were infected with *L. donovani* i.v. 16h previously. IFNγ responses of splenic iNKT cells from naïve and infected B6 and CD47$^{-/-}$ mice (n=5 mice per group) are shown as percentage of IFNγ^{+} cells (corrected for isotype staining) following detection with CD1d tetramer in addition to surface labeling of CD3 (open bars), or surface and intracellular labeling of CD3 (grey bars). Statistical analysis was performed with a one-way ANOVA ***, P <0.0001, NS = not significant.
